# Emerging Role of Hepatic Ketogenesis in Fatty Liver Disease

**DOI:** 10.3389/fphys.2022.946474

**Published:** 2022-07-04

**Authors:** Raja Gopal Reddy Mooli, Sadeesh K. Ramakrishnan

**Affiliations:** Division of Endocrinology and Metabolism, Department of Medicine, University of Pittsburgh, Pittsburgh, PA, United States

**Keywords:** Hepatic ketogenesis, NAFLD, NASH, HCC, HMGCS2

## Abstract

Non-alcoholic fatty liver disease (NAFLD), the most common chronic liver diseases, arise from non-alcoholic fatty liver (NAFL) characterized by excessive fat accumulation as triglycerides. Although NAFL is benign, it could progress to non-alcoholic steatohepatitis (NASH) manifested with inflammation, hepatocyte damage and fibrosis. A subset of NASH patients develops end-stage liver diseases such as cirrhosis and hepatocellular carcinoma. The pathogenesis of NAFLD is highly complex and strongly associated with perturbations in lipid and glucose metabolism. Lipid disposal pathways, in particular, impairment in condensation of acetyl-CoA derived from β-oxidation into ketogenic pathway strongly influence the hepatic lipid loads and glucose metabolism. Current evidence suggests that ketogenesis dispose up to two-thirds of the lipids entering the liver, and its dysregulation significantly contribute to the NAFLD pathogenesis. Moreover, ketone body administration in mice and humans shows a significant improvement in NAFLD. This review focuses on hepatic ketogenesis and its role in NAFLD pathogenesis. We review the possible mechanisms through which impaired hepatic ketogenesis may promote NAFLD progression. Finally, the review sheds light on the therapeutic implications of a ketogenic diet in NAFLD.

## Ketone Bodies- Role for Energy Fuel and Cellular Signaling

The ketone bodies, namely acetoacetate (AcAc), acetone, and β-hydroxybutyrate (βOHB), are small lipid-derived metabolites that acts as an alternative form of energy for all forms of life (Hasselbalch et al., 1994). The levels of ketone bodies, AcAc and βOHB are abundant compared to acetone (Laffel, 1999). Under physiological conditions, ketone bodies contribute 5–20% of total energy metabolism (Cox et al., 2016). Ketone body generation and utilization are influenced by various physiological cues, including nutrient deprivation, exercise, and calorie restriction, where their serum concentrations could rise from 100-250μM to 1 mM (Fery and Balasse, 1983; Fèry and Balasse, 1988; Balasse and Féry, 1989). Notably, ketone body levels also peaks postnatal (10–15 days after birth) and reaches to 2–3 mM to support the huge energy demands of developing neonates (Bougneres et al., 1986; Arima et al., 2021). Short-term exposure to a fat-enriched diet, such as high-fat diet (HFD), also increases circulatory ketone bodies (Satapati et al., 2008; Sunny et al., 2010). Moreover, a ketogenic low-carb HFD increases serum ketone body levels above 2 mM (Gano et al., 2014; Ulamek-Koziol et al., 2019a). Elevated ketone bodies are also found in pathological conditions such as uncontrolled diabetes and alcoholic ketoacidosis, where the levels reach as high as 20 mM) (Fery and Balasse, 1985; Mitchell et al., 1995). However, the role of ketone bodies in pathological conditions remains to be elucidated.

In addition to serving as fuel, ketone bodies act as a metabolic signal regulating diverse cellular functions (Newman and Verdin, 2014). βOHB but not acetone or AcAc signals through the G-protein-coupled receptors (GPR), namely GPR109A, also known as the niacin receptor (HCAR2). GPR109A is highly expressed in adipose tissue and immune cells (Singh et al., 2014). GPR109A signaling in adipose tissue inhibits hormone-sensitive lipase-mediated lipolysis via repression of adenylyl cyclase. This has been proposed to play a critical role in inhibiting lipolysis in the adipose tissue, perhaps as a feedback mechanism to decrease ketone body synthesis by limiting the free fatty acid supply (Taggart et al., 2005). βOHB signaling via GPR109A also regulates inflammation via NLRP3 (NOD-,LRR-and pyrin domain-containing protein 3) (Macia et al., 2015), reverse cholesterol transport (Wu and Zhao, 2009), atherosclerosis (Zhang et al., 2021) and neuroprotection (Rahman et al., 2014). Ketone bodies also signal through the free fatty acid receptor (FFAR3), also known as GPR41, which was initially identified as a receptor for short-chain fatty acids (SCFAs). Under ketogenic conditions, activation of GPR41 in sympathetic ganglions suppresses energy expenditure (Kimura et al., 2011; Won et al., 2013; Miyamoto et al., 2019). Thus, ketone bodies reduce lipolysis, sympathetic activity, and overall metabolic rate *via* GPRs.

βOHB is structurally similar to butyrate, which acts as an endogenous inhibitor of class I histone deacetylases (HDACs), the enzyme that deacetylates histone and non-histone proteins (Cousens et al., 1979; Kong et al., 2017). Shimazu and colleagues demonstrated that βOHB inhibits class I HDACs *in vitro* with an IC_50_ of 2.4–5.3 mM, while AcAc inhibits class I HDACs at a higher concentration (Shimazu et al., 2013). Consistent with the role of ketone bodies in repressing HDACs, elevating ketone bodies *via* exogenous administration, fasting, or calorie deprivation increases the global histone acetylation marks on the chromatin (Shimazu et al., 2013; Gensous et al., 2019). Thus, ketone bodies, via epigenetic mechanism, regulate the expression of several genes involved in anti-oxidant and anti-inflammatory response (Youm et al., 2015; Rojas-Morales et al., 2020).

## Hepatic Ketogenesis

**FIGURE 1:**
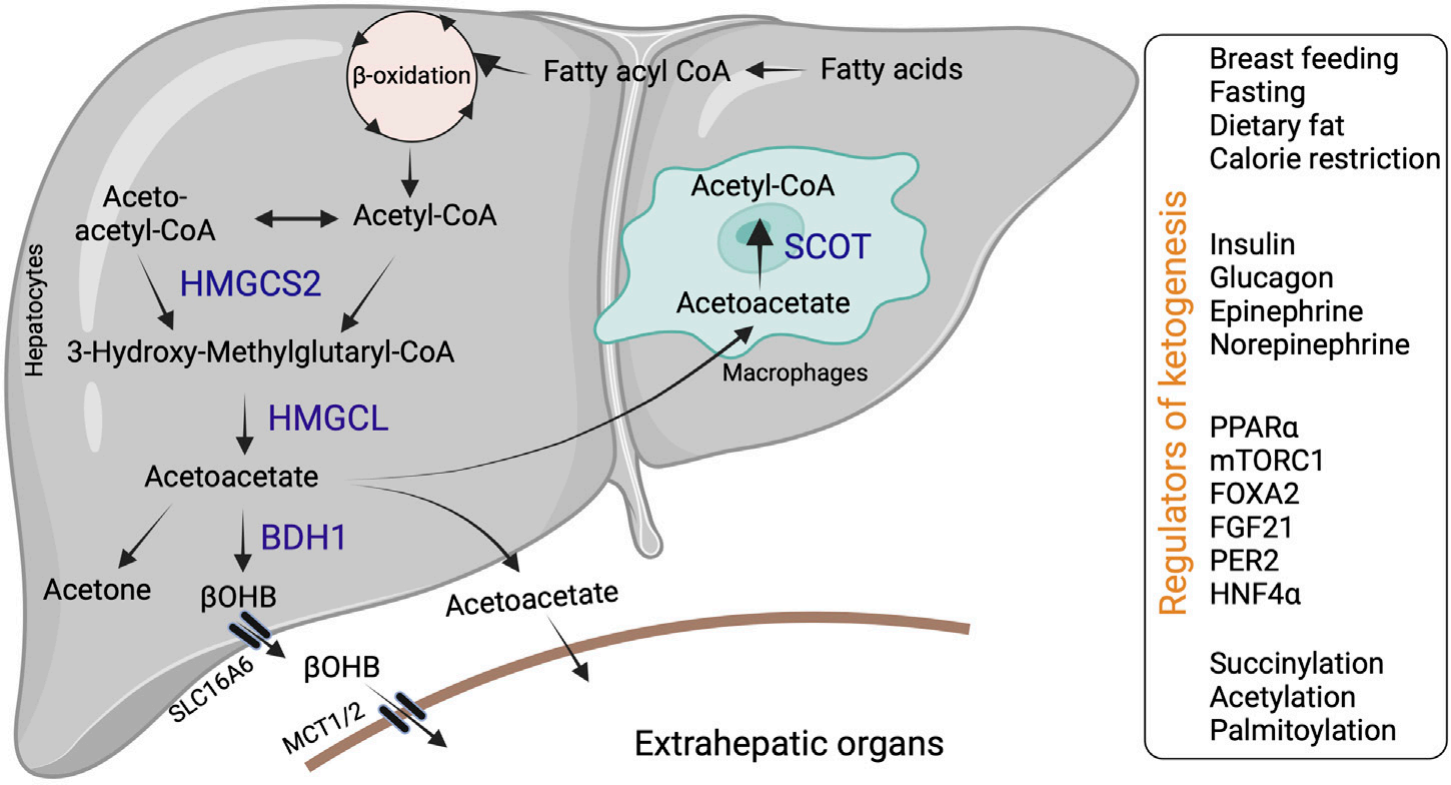
Hepatic ketone body generation, utilization and its regulators. Ketone bodies are primarily synthesized in the hepatocytes through sequential enzymatic reactions. Ketone bodies released from the hepatocytes are taken up by extrahepatic tissues via specific transporters and metabolized with the help of SCOT. Additionally, liver macrophages could metabolize acetoacetate with the help of SCOT. Hepatic ketogenesis is regulated by dietary fat, physiological cues and at transcriptional and translational levels. Abbreviations: BDH1, β-hydroxybutyrate dehydrogenase; HMGCL-HMG-CoA lyase; HMGCS2- 3-hydroxy-3-methylglutaryl-CoA synthase 2; βOHB-β-hydroxybutyrate; SCOT-Succinyl-CoA:3-ketoacid coenzyme A transferase MCT1-monocarboxylic acid transporter; SLC16A6-solute carrier family16, member 6.

Ketogenesis occurs through a series of enzymatic reactions, wherein acetyl-CoA derived from the fatty acid β-oxidation is condensed to acetoacetyl-CoA via acetoacetyl-CoA thiolase in the mitochondrial matrix (Williamson, 1985). Acetoacetyl-CoA is converted to hydroxymethyl glutaryl-CoA (HMG-CoA) by the mitochondrial rate-limiting ketogenic enzyme 3-hydroxy-3-methylglutaryl-CoA synthase 2 (HMGCS2, EC 2.3.3.10) (Hegardt, 1999). HMG-CoA lyase (HMGCL, EC 4.1.3.4) then cleaves HMG-CoA to liberate acetyl-CoA and acetoacetate. β-OHB is generated from acetoacetate by the phosphatidylcholine-dependent mitochondrial enzyme D-βOHB dehydrogenase (BDH1, EC 1.1.1.30) (Figure 1). β-OHB is considered the most stable isoform and abundant circulating ketone body, while AcAc can also impulsively decarboxylate to acetone (Williamson and Whitelaw, 1978; Heitmann et al., 1987; Fukao et al., 2004).

In mammals, ketogenesis primarily occurs in the liver due to the abundant expression of the HMGCS2 in the hepatocytes (Hegardt, 1999). Interestingly, hepatocytes do not express the ketolytic mitochondrial enzyme succinyl-CoA:3-oxo-acid CoA-transferase (SCOT, EC 2.8.3.5). Thus, hepatocytes only generate ketone bodies but cannot oxidize them (Fukao et al., 1997). Ketone bodies are exported from the hepatocytes via the solute carrier family 16, member 6 (SLC16A6). Ketone body uptake in the target tissue occurs through monocarboxylate transporters (MCT1/2) (Figure 1). The brain and heart are the primary users of ketone bodies (Owen et al., 1967; Sokoloff, 1973; Abdul Kadir et al., 2020; Lopaschuk et al., 2020), though a small amount is utilized by other organs (Wang et al., 2019a; Cheng et al., 2019; Cuenoud et al., 2020; Tomita et al., 2020). The ketone bodies are oxidized into acetyl-CoA (Pellerin et al., 2005). The acetyl-CoA enters into the TCA cycle or lipogenesis or is excreted in the urine (Fukao et al., 2004).

The second highest expression of HMGCS2 is observed in the intestinal epithelial cells (Cheng et al., 2019). A recent study showed that a loss of HMGCS2 in intestinal stem cells compromises their ability to differentiate and regenerate (Wang et al., 2017; Cheng et al., 2019). Though, HMGCS2 expression is thought to be negligible in other mammalian cells; recent evidence shows that retinal pigment epithelium (Adijanto et al., 2014), kidney (Zhang et al., 2011; Takagi et al., 2016), heart (Shukla et al., 2017), astrocytes (Le Foll et al., 2014; Grabacka et al., 2016a; Thevenet et al., 2016), skeletal muscle (Crooks et al., 2014), pancreatic β-cells (El Azzouny et al., 2016), and beige adipocytes express HMGCS2 and produce ketone bodies in small amounts (Wang et al., 2019a). Moreover, pathological conditions such as diabetes, kidney diseases and cardiovascular diseases induce HMGCS2 expression in extrahepatic tissues (Zhang et al., 2011; Shukla et al., 2017). But what remain unclear is the contribution of extrahepatic tissues to systemic ketone body levels. A recent study using liver specific-HMGCS2 knock-out mice demonstrated that the circulatory ketone bodies are derived from the liver (Venable et al., 2022). Thus, the extrahepatic tissues are speculated to have no contribution to the circulating pool of ketone bodies under steady state. Whether the ketone bodies exert local effect in the target tissue remains unclear.

## Regulation of Hepatic Ketogenesis

### Nutritional Regulation

Hepatic ketogenesis is regulated by nutritional and physiological cues (Balasse and Féry, 1989; Kolb et al., 2021; Koronowski et al., 2021). For instance, the postnatal increase in hepatic ketogenesis is attributed to a surplus of dietary fat from breast milk (Asif et al., 2022). Thus, early weaning of mice reduces serum ketone levels due to a decrease in dietary fat (Asif et al., 2022). Similarly, HFD-mediated increase in serum fatty acids induces hepatic ketogenesis and elevates serum ketone body levels (Sunny et al., 2010). These data partially denote that dietary fatty acids act as primary substrates for hepatic ketogenesis. Not surprisingly, mobilization of free fatty acids from the adipose tissue is directly proportional to hepatic ketogenesis (Fougerat et al., 2021). There exists a concept of a precursor-product relationship between total fat oxidation and hepatic ketogenesis (McGarry and Foster, 1980). Adipose tissue lipolysis elevates serum free fatty acids (McGarry and Foster, 1980; Arner, 2002; Morigny et al., 2016). Studies show that inhibiting adipose tissue lipolysis by disrupting adipose triglyceride lipase (ATGL) abrogates increasing serum ketone bodies suggesting that adipose tissue-derived fatty acids are necessary for hepatic ketogenesis (Jaeger et al., 2015; Schreiber et al., 2017). However, it remains unclear whether ATGL inhibition impact hepatic ketogenesis in diet-induced obesity, where circulating fatty acids are elevated. Conflicting data also show that free fatty acids can be elevated *in vivo* without an increase in the ketone bodies (Felts and Mayes, 1965; Krebs et al., 1969). Inversely, ketosis could be reversed in situations of elevated serum free fatty acids (McGarry and Foster, 1971; McGarry et al., 1973). Therefore, these *in vivo* studies indicate that the rate of hepatic ketogenesis is not dependent solely upon the substrate availability i.e., fatty acids. Moreover, it is also possible that the continuous accumulation of fatty acids in the liver could potentially induce oxidative, mitochondrial stress, and even insulin resistance (Schoiswohl et al., 2015), which can impact ketogenesis. Seemingly, the hormonal, transcriptional, and post-translational modifications in the liver coordinate the maximal rate of ketone body synthesis (Figure 1) (Willms et al., 1969).

### Hormonal and Molecular Regulators of Hepatic Ketogenesis

Various physiological cues regulate hepatic ketogenesis through diverse mechanisms at hormonal, transcriptional, and post-translational levels (Grabacka et al., 2016b). For example, the expression and activity of HMGCS2 is regulated by insulin and glucagon (Alberti et al., 1978). Insulin inhibits hepatic ketogenesis by suppressing HMGCS2 expression in the liver and limiting substrate availability *via* reducing adipose tissue lipolysis (Chakrabarti et al., 2013). Conversely, glucagon promotes HMGCS2 expression via the transcription factor peroxisome proliferator-activated receptor alpha (PPARα) and increases the ketogenic flux of fatty acids (Liljenquist et al., 1974; Cotter et al., 2014a). Other hormones, such as epinephrine and norepinephrine, also activate ketogenesis by stimulating lipolysis (Keller et al., 1989; Beylot, 1996). At the transcriptional level (Hegardt, 1998), PPARα family of transcription factors regulate *Hmgcs2* expression in various tissues (Rodríguez et al., 1994; Mihaylova et al., 2018). In the liver, PPARα is the primary regulator of *Hmgcs2* and ketogenesis (Meertens et al., 1998). Thus, mechanisms that regulate PPARα transcriptional activity in the liver modulates hepatic ketogenesis. For instance, PPARα transcriptional activity is inhibited by the mammalian target of rapamycin complex 1 (mTORC1), resulting in the suppression of *Hmgcs2* expression and ketogenesis (Sengupta et al., 2010). In intestine stem cells and colonocytes, PPARα and PPARγ regulate *Hmgcs2* expression, respectively (Kim et al., 2019a; Cheng et al., 2019; Mana et al., 2021). Other transcription factors, such as forkhead box 2 (FOXA2) are also shown to regulate *Hmgcs2* transcription (Nakamura et al., 2007). Similarly, the circadian expression of HMGCS2 is regulated by the liver period 2 (PER2) via an unknown mechanism (Chavan et al., 2016). In addition to positive regulators, several transcription factors act as negative regulators of ketogenesis. For example, hepatocyte nuclear factor 4 (HNF4) represses *Hmgcs2* expression (Figure 1) (Rodríguez et al., 1998).

The post-translational modifications such as succinylation, acetylation, and palmitoylation regulate HMGCS2 enzyme activity (Figure 1) (Stram and Payne, 2016). Shimazu *et.al* demonstrated that acyltransferases acetylate HMGCS2 at Lys 310, 447 and 473 (Shimazu et al., 2010). Using genetic *in vivo* models, the authors showed that deacetylation of HMGCS2 by sirtuin 3 (SIRT3), which belongs to the deacetylase/ADP-ribosylase family of sirtuins, increases HMGCS2 enzyme activity. SIRT3 also activates the enzymes involved in fatty acid oxidation, such as LCAD, contributing to the induction of hepatic ketogenesis (Shimazu et al., 2010). Succinylation also represses HMGCS2 activity by binding to and competitively inhibiting the active site. For example, Quant *et.al* showed that the attachment of succinyl-CoA to the catalytic cysteine residue on HMGCS2 blocks the binding of acetoacetyl-CoA to the substrate. Glucagon enhances HMGCS2 enzyme activity by decreasing the levels of succinyl-CoA (Quant et al., 1990). It is interesting to note that the enzymes involved in the generation of ketone bodies are heavily succinylated. In particular, Hmgcs2 is succinylated at least on 15 lysine residues (Quant et al., 1989; Rardin et al., 2013). Conversely, the post-translational modification via palmitoylation has been shown to increase HMGCS2 enzyme activity (Kostiuk et al., 2008; Kostiuk et al., 2010). Thus, the enzymatic activity of HMGCS2 is regulated through post-translational modifications under both physiological and pathological conditions.

## Hepatic Ketogenesis and Non-Alcoholic Fatty Liver

Non-alcoholic fatty liver (NAFL), also known as simple steatosis, begins with the accumulation of triglycerides in the form of lipid droplets in the cytoplasm of hepatocytes (Friedman et al., 2018; Loomba et al., 2021). This occurs in response to increased lipid acquisition through diet, *de novo* lipogenesis (DNL), and fatty acid mobilization from peripheral tissues. For example, continuous delivery of non-esterified fatty acids (NEFAs) to the liver through adipose tissue lipolysis provides the substrate for the synthesis of intrahepatic triglycerides (IHTG) (Donnelly et al., 2005; Lomonaco et al., 2012). As compensation for the large influx of lipids, mitochondrial β-oxidation, a critical oxidative pathway for the disposal of NEFAs is upregulated. This results in the accumulation of acetyl-CoA, which has two fates; either undergo oxidation through the tricarboxylic acid (TCA) cycle or condense in the ketogenic pathway to form ketone bodies. Ketogenesis disposes of as much as three-fold fat entering the liver (Satapati et al., 2015; Grattagliano et al., 2019). Therefore, dysregulation in the ketogenesis results in a flux of Acetyl-CoA into the lipogenic pathway, contributing to NAFL pathogenesis (Cotter et al., 2014b).

### Status of Ketogenesis in Non-Alcoholic Fatty Liver

**FIGURE 2:**
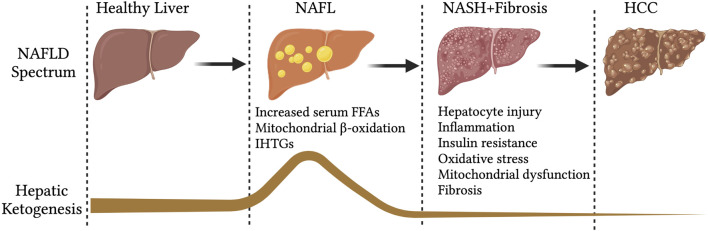
Hepatic ketogenesis status during the development of NAFLD to HCC. During the spectrum of NAFLD, the hepatic ketogenesis was augmented in response to entry of fatty acids into the liver. However, as the NAFLD progress to NASH, fibrosis and HCC, the ketogenesis was significantly impaired. Abbreviations: NAFLD-non-alcoholic fatty liver disease, NAFL-non-alcoholic fatty liver, IHTGs-intrahepatic triglycerides, NASH-non-alcoholic steatohepatitis, HCC-Hepatocellular carcinoma.

**FIGURE 3:**
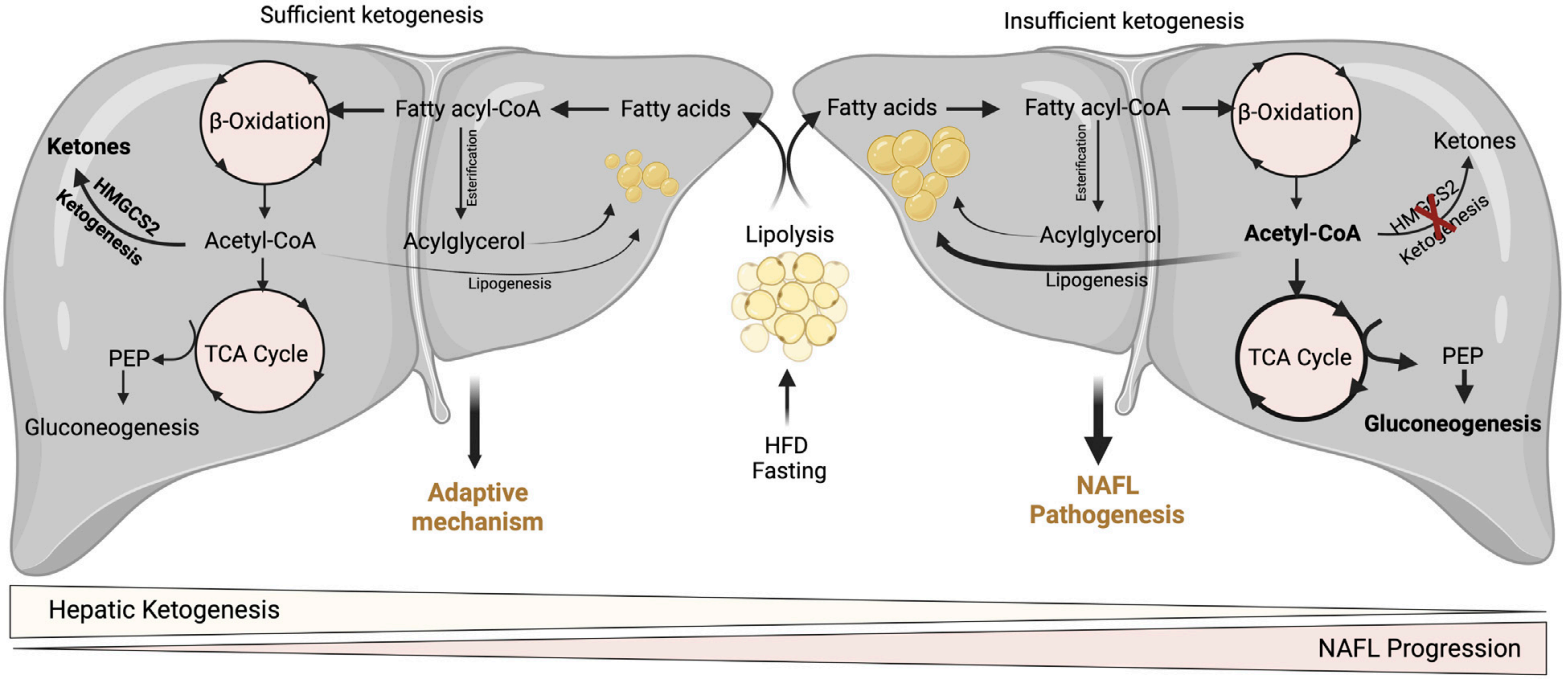
Adaptive and pathological significance of hepatic ketogenesis in NAFL. Under hepatic ketogenesis sufficient conditions, the increased fatty acid entry into the liver during fasting and HFD can efficiently enter into ketogenesis pathway and protects from excessive accumulation of lipids and thereby lipotoxicity. On the other hand, under insufficient hepatic ketogenesis condition, the excess fatty acids result in the accumulation of acetyl-CoA and enter to TCA cycle, and thereby increasing gluconeogenesis and DNL. Thus, continuous accumulation of fatty acids results in lipotoxicity and NAFL progression.

Hepatic mitochondrial fatty acid β-oxidation is augmented in NAFL (Koliaki and Roden, 2013; Koliaki et al., 2015), when it is not associated with any degenerative liver function, insulin resistance or cardiovascular diseases (Bickerton et al., 2008). In line with that, hepatic ketogenesis and even circulatory ketone bodies are increased in humans and mouse models of NAFL (Figure 2) (Sunny et al., 2010; Satapati et al., 2012). This phenomenon is likely to occur during fasting and short-term exposure to HFD (Figure 2). During fasting, hepatic fatty acid β-oxidation is induced by the ligand-activated transcription factor PPARα (Yoon, 2009; Mooli et al., 2021), which transcriptionally activates hepatic ketogenesis (Sengupta et al., 2010). Similarly, exposure to mice HFD for 16-weeks induced PPARα-mediated fatty acid β-oxidation and hepatic ketogenesis (Sunny et al., 2010). The speculation from these observations is that hepatic ketogenesis reflects a metabolic compensation for the increased delivery of fatty acid to the liver, helping to offload the hepatic fat (Solinas et al., 2015). A study using magnetic resonance spectroscopic methods in animals of NAFL with moderate insulin resistance revealed an increased ketone turn-over rate (Satapati et al., 2012) (Figure 3). Thus, hepatic ketogenesis reflects NAFL as long as other hepatic features remain clinically normal.

### Do Ketogenic Insufficiency Promote Non-Alcoholic Fatty Liver?

A wealth of studies has revealed the significance of hepatic ketogenesis on lipid metabolism in NAFL in both murine and human models (Inokuchi et al., 1992; Fukao et al., 2004; Puisac et al., 2018; Sikder et al., 2018; Lee et al., 2019; Mey et al., 2020). For example, deletion of hepatic *Hmgcs2* using antisense oligonucleotides resulted in a significant increase in hepatic neutral lipid accumulation following regular chow and high-fat feeding conditions in both neonates and adult mice (Cotter et al., 2014b; d'Avignon et al., 2018). Moreover, a recent study has shown that global and hepatocyte-specific disruption of *hmgcs2* results in a massive deposition of hepatic triglycerides in neonates (Arima et al., 2021). The underlying mechanisms through which hepatic ketogenic insufficiency promotes lipid accumulation is poorly defined. Nevertheless, it has been demonstrated that increased TCA cycle flux and DNL from acetyl-CoA may contribute to the steatosis upon impaired ketogenesis (Cotter et al., 2014b). Studies show that ketogenesis-insufficiency increases the expression of several DNL-related genes such as *Srebp1*, *Chrebp1*, and *Scd2* (Asif et al., 2022; Cotter et al., 2014b; d'Avignon et al., 2018). The other mechanism by which hepatic ketogenic insufficiency promotes lipid accumulation is through mitochondrial stress/dysfunction. For example, a recent study showed that hepatic ketogenic insufficiency in neonates increases acetylation of mitochondrial proteins involved in oxidative phosphorylation resulting in reducing their enzymatic activity (Arima et al., 2021). Thus, hepatic ketogenesis plays a critical role in NAFL by regulating the portioning of acetyl-CoA towards lipogenesis, and mitochondrial dysfunction. It is also well established that an increased rate of gluconeogenesis is strongly associated with high IHTG (Fabbrini et al., 2008; Sunny et al., 2011). For instance, impairment in ketogenesis results in the accumulation of acetyl-CoA in the mitochondria and thus diverts towards gluconeogenesis (Cotter et al., 2014b) (Figure 3). In addition to ketogenic insufficiency role in NASH progression, a recent study has shown that overexpression of Hmgcs2 in mice showed a significant reduction in hepatic lipids (Asif et al., 2022). Thus, the study suggests the activation of hepatic ketogenesis may serve as a therapeutic strategy for alleviating NAFL and NASH.

## Hepatic Ketogenesis and Non-Alcoholic Steatohepatitis

NAFL is considered self-limited; however, it can progress to non-alcoholic steatohepatitis (NASH) (Kawano and Cohen, 2013; Goh and McCullough, 2016; Mooli and Ramakrishnan, 2022a). NASH is characterized by hepatocyte ballooning and cell death, inflammatory cell infiltration, and collagen deposition (fibrosis) (Machado and Diehl, 2016; Koyama and Brenner, 2017; Mooli and Ramakrishnan, 2022b). A “two-hit” theory explains the mechanism of NASH pathogenesis, wherein simple fat accumulation in the hepatocytes (first hit) converts to lipotoxicity (second hit) with increased levels of free fatty acids, cholesterol and other lipid metabolites (Buzzetti et al., 2016). Consequently, this results in mitochondrial dysfunction with reactive oxygen species (ROS) production and endoplasmic reticulum (ER) stress (Pérez-Carreras et al., 2003; Browning and Horton, 2004). The mitochondrial β-oxidation determines lipotoxicity in the liver, because it acts as the dominant oxidative pathway for disposal of excess fatty to either through oxidation via TCA cycle or ketogenic pathway in the liver (Morris et al., 2011; Serviddio et al., 2011). Thus, during NAFL to NASH progression, the hepatic oxidative metabolism increases instead of removing the excess fatty acids via ketogenic pathway (Rolo et al., 2012).

### Status of Ketogenesis in Non-Alcoholic Steatohepatitis

**FIGURE 4:**
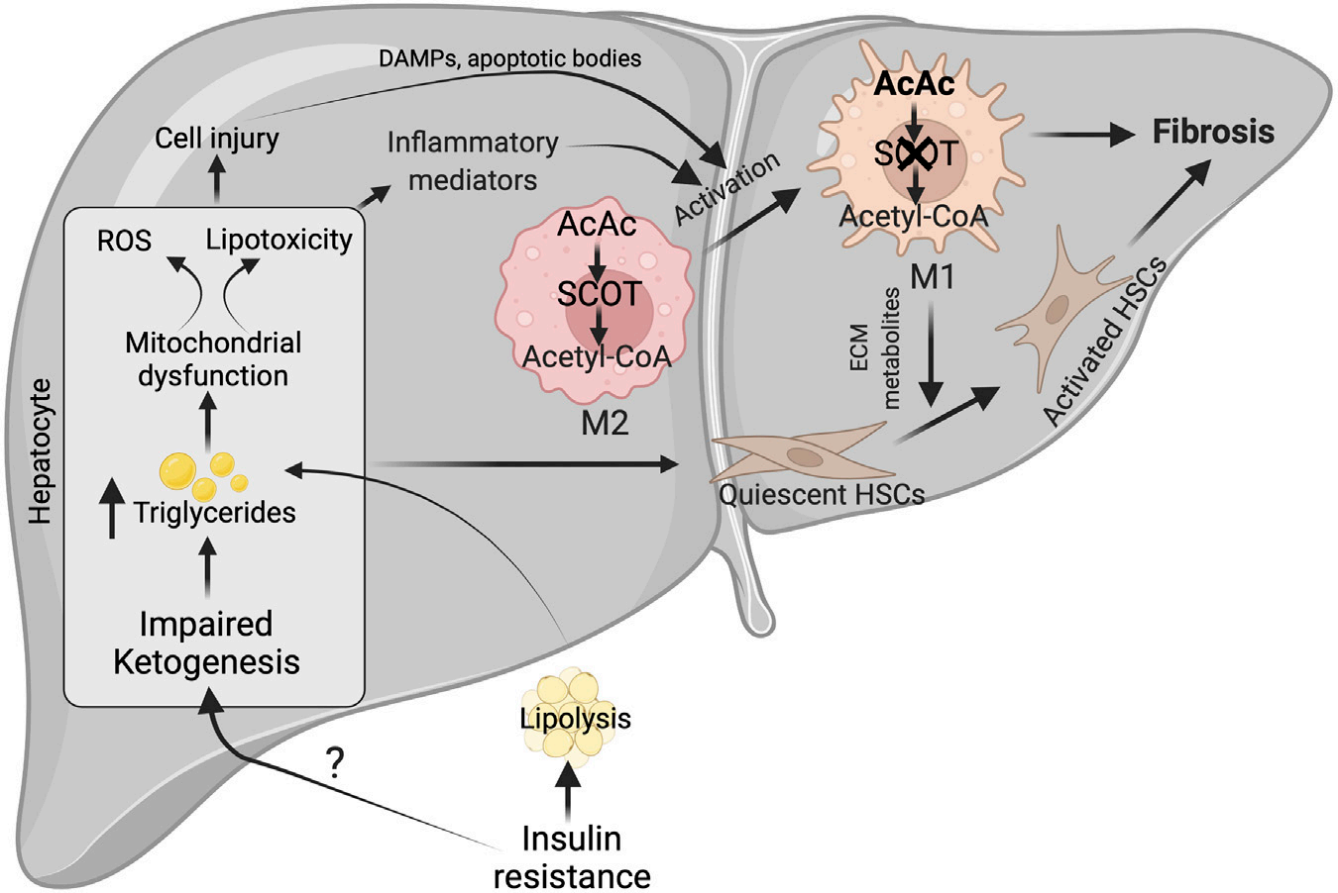
Possible mechanisms for impairment of hepatic ketogenesis and its contribution to NASH and fibrosis. Under NASH, insulin resistance was the major contributing factor for the impairment of hepatic ketogenesis. This impairment in hepatic ketogenesis can promote lipotoxicity, mitochondrial dysfunction and ROS generation in the hepatocytes. Finally, all these results in NASH progression by activating cell injury and inflammation. In addition, the impairment in hepatic ketogenesis can promote fibrosis through the various factors derived from the injured hepatocytes and activation of macrophages. The activation of macrophages takes place when there is an impairment in the oxidation of AcAc, resulting in the accumulation of acetyl CoA. This results in conversion of M2 macrophage phenotype to M1, which are pro-inflammatory in nature. ROS-reactive oxygen species, AcAc-acetoacetate, SCOT-succinyl-CoA-oxoacid transferase, ECM-extracellular matrix.

Unlike NAFL, NASH patients display impaired hepatic ketogenesis reflected by a reduction in circulatory ketone body levels (Männistö et al., 2015). Importantly, obese patients with fatty liver have reduced total ketone body levels compared to obese patients without fatty liver. Serum βOHB negatively correlates with liver fat and positively with insulin sensitivity in obesity-related NAFLD patients (Mey et al., 2020). This is evident in obese NAFLD patients with impaired insulin sensitivity, hyperlipidemia, and liver injury, having lower circulating ketone bodies (Fletcher et al., 2019). Further, these patients show resistance to ketosis induced by a 24 h fast (Fletcher et al., 2019). Similarly, chronic exposure of mice with HFD for 32 weeks results in a significant decrease in serum ketone levels due to a reduction in hepatic *Hmgcs2* expression (Asif et al., 2022). Although the reason for the discrepancy in hepatic ketogenesis under moderate and chronic HFD exposure is not well defined, accumulating evidence shows a link between insulin resistance and inflammation (Figure 4). In support, hepatic ketogenesis is found to be augmented initially with HFD (Sunny et al., 2010) when insulin levels are higher, and insulin sensitivity is intact. However, prolonged exposure to HFD progressively decrease ketogenesis (Asif et al., 2022), which is temporally linked with insulin resistance and compounded by other conditions such as oxidative stress and mitochondrial damage (Mooli et al., 2022). But how insulin resistance affects hepatic ketogenesis? Insulin resistance decreases hepatic ketogenesis by two ways. Firstly, some of the metabolic effects of insulin are retained under insulin resistance. For example, insulin triggers DNL, which attenuates mitochondrial NEFA transport and β-oxidation necessary for hepatic ketogenesis (McGarry et al., 1978). Second, mTORC1 activation or increased TCA flux via enhancing FOXO targets suppresses hepatic ketogenesis (Kucejova et al., 2016). The constitutive activation of mTORC1 during insulin resistance may also mediate the paradoxical activation of lipid synthesis through Srebp activation (Li et al., 2010). The other possible mechanism by which mTORC1 suppresses hepatic ketogenesis is through repression of Pparα activity (Sengupta et al., 2010). Reduced hepatic fatty acid oxidation were also noted in leptin receptor-deficient rats due to strong induction of mTORC1 signaling (Zheng et al., 2009) and (Satapati et al., 2008). Thus, repression of hepatic ketogenesis may precede insulin resistance in NASH through mTORC1 signaling pathways (Satapati et al., 2012) (Kucejova et al., 2016). Overall, ketogenesis is downregulated in NASH through mechanisms that remain incompletely understood.

### Do Ketogenic Insufficiency Promote Non-Alcoholic Steatohepatitis and Fibrosis?

Although it is unclear how the severity of NASH impairs hepatic ketogenesis, it is well established that hepatic ketogenic insufficiency is associated with NASH and fibrosis. Cotter *et. al* showed that HFD feeding to mice with hepatic ketogenic insufficiency induces severe hepatocellular injury, characterized by an increased number of sinusoidal macrophages, infiltration of inflammatory cells, and accumulation of dying hepatocytes. These results suggest that hepatic ketogenic insufficiency accelerates NASH-like phenotype upon overnutrition (Cotter et al., 2014b). The authors also observed an increase in the anaplerotic flux of acetyl-CoA through TCA cycle (Cotter et al., 2014b), which lead to higher ROS generation (Murphy, 2009; Zhao et al., 2017). Elevated ROS causes hepatocellular damage by inducing DNA damage and increasing the accumulation of toxic lipids and proteins (Rolo et al., 2012; Masarone et al., 2018). In support of this concept, Xu *et al.* demonstrated that the increase in NASH characteristics upon Bdh1 knockdown is associated with increased ROS levels (Xu et al., 2022). Conversely, *Bdh1* overexpression successfully attenuated lipotoxicity, oxidative stress-induced hepatic injury, inflammation and apoptosis in the fatty liver from db/db mice (Xu et al., 2022). Additionally, several lines of evidence support the concept that hepatic ketogenesis might regulate NASH phenotype through mitochondrial metabolism and inflammation. For example, βOHB inhibits NLRP3 inflammasome activation in macrophages (Youm et al., 2015; Kim et al., 2019b). Moreover, βOHB has been shown to improve the resistance to oxidative stress (Wei et al., 2014; Oka et al., 2021). Based on this concept, a recent study has hypothesized that activation of ketogenesis in the liver could potentially attenuate ROS-mediated NASH progression (Figure 4). Thus, the studies suggest that impairment in hepatic ketogenesis makes the liver more susceptible to NASH.

The other characteristic feature of NASH is fibrosis (Schwabe et al., 2020). The activation of resident hepatic stellate cells into myofibroblasts to produce matrix proteins is the crucial step in fibrogenesis (Mederacke et al., 2013). The liver macrophages and apoptotic or injured hepatocytes play a critical role in hepatic fibrogenesis by releasing several cytokines and growth factors (Lech and Anders, 2013; An et al., 2020). The plasticity of macrophages phenotype i.e. classically polarized M1 macrophages promotes stellate cell activation, while alternatively polarized M2 macrophages functions differently (Sica et al., 2014; Bai et al., 2017). Recent studies have shown that macrophage plasticity is determined by intrinsic metabolism, including fatty acid oxidation, glycolysis, and ketone body oxidation (Newsholme et al., 1986; Nomura et al., 2016; Liu et al., 2021). But the question is how relevant is hepatic ketogenesis in fibrosis. The present data show that an impairment in hepatic ketogenesis induces fibrosis in two ways. First, impairment in ketogenesis increases lipid accumulation in the hepatocytes resulting in lipotoxicity and cell apoptosis (Cotter et al., 2014b). During this process, factors derived from the injured hepatocytes activate hepatic stellate cells either directly or via the macrophages (Hirsova et al., 2016; Geng et al., 2021). Second, mitochondrial enzyme SCOT, which oxidizes ketone bodies, is abundantly expressed in the macrophages (Youm et al., 2015). Puchalaska et al. have shown that impairment in the oxidation of AcAc in the hepatic macrophages is associated with an accelerated fibrotic response via trans-differentiation of hepatic stellate cells into myofibroblasts (Puchalska et al., 2019) (Figure 4). This is evident in mice with hepatic ketogenesis insufficiency, where stellate cell activation and fibrosis are augmented (Cotter et al., 2014b). Thus, an impairment of hepatic ketogenesis and ketone body metabolism in the macrophages converge in inducing liver fibrosis.

## Hepatic Ketogenesis and Hepatocellular Carcinoma

Hepatocellular carcinoma (HCC) is one of the most common liver malignancies and a second leading cause of cancer-related death. HCC accounts for nearly 70–85% of all liver cancers (Balogh et al., 2016). Growing evidence suggests NAFL or NASH are the primary risk factors for HCC, although chronic hepatitis is also among the other risk factors (Anstee et al., 2019; Huang et al., 2021). In particular, NAFLD with advanced fibrosis or cirrhosis increases the susceptibility to HCC (McPherson et al., 2015). The progression of NASH to HCC is largely associated with the change in metabolism, including lipogenesis, fatty acid oxidation and glycolysis (Feng et al., 2020; Chen et al., 2021; Ren et al., 2021). This raises the question of the status and the contribution of hepatic ketogenesis in HCC.

Studies show that the expression of HMCS2 is decreased in the livers of patients with nodular cirrhosis and HCC patients. Further, the decreased expression was associated with higher pathological grades and clinical stages of HCC (Wang et al., 2019b) (Figure 2). In human HCC cell lines, knockdown of HMGCS2 increases tumor growth and cell migration, while HMGCS2 overexpression decreases cell proliferation and increases apoptosis (Wang et al., 2019b; Wang et al., 2020). Further, exogenous ketone bodies inhibit the proliferation of HCC cells and their invasiveness (Wang et al., 2019b). Mechanistically, HCC progression upon HMGCS2 deletion is associated with increased fatty acid and cholesterol biosynthesis in HCC cell lines. Future studies with genetic mouse models are needed in addressing the contribution of hepatic ketogenesis in HCC development and progression.

## Ketogenic Diet and Non-Alcoholic Fatty Liver Disease

NAFLD management is crucial to counteract its increasing prevalence. Although there are no effective drug targets, lifestyle and dietary modifications effectively improve NAFLD (Thoma et al., 2012; Mahesh et al., 2016; Raja Gopal Reddy et al., 2016; Mahesh et al., 2017; Paris et al., 2017). For example, energy-deficient metabolic states, such as calorie restriction and intermittent fasting, alleviates hepatic steatosis (Johari et al., 2019; Mooli et al., 2020; Holmer et al., 2021). In addition, diets low in carbohydrates significantly reduce the IHTG content (York et al., 2009; Holmer et al., 2021).These dietary regimens alleviate hepatic lipids by lowering body weight and insulin resistance (Volek et al., 2009; Skytte et al., 2019). Similarly, ketogenic diet (KD), composed of high-fat, very low carbohydrate and adequate proteins has been used since the early 1920s to control seizures in patients with epilepsy (Peterman, 1928; Ulamek-Koziol et al., 2019b). In recent decades, KD has received extensive attention because of its beneficial effects on various diseases, including obesity, type 2 diabetes, heart disorders, cancers, and intestinal disorders (Veech, 2004; Zhu et al., 2022). Several modified KD were formulated, including the classic long-chain triglycerides (LCT) KD, the modified Atkins diet (MAD), the medium-chain triglyceride oil diet (MCT), low glycemic index treatment (LGIT), and intermittent fasting (IF) (Zhu et al., 2022). The classical LCT KD is the most used KD that incorporates a 4:1 ratio of fat (in grams) to proteins plus carbohydrate (in grams) (Hassan et al., 1999; Coppola et al., 2002). The unpalatable nature of LCT KD led to the development of MCT KD, which is more acceptable and ketogenic than LCTs (Huttenlocher et al., 1971). The MCT KD is not based on diet ratios but rather uses a percentage of calories from MCT oil to create ketones, and it is shown to be frequently associated with gastrointestinal side effects such as diarrhea, vomiting, and bloating (Liu and Wang, 2013). Similarly, MAD KD is based on the Atkins diet that shares similar food composition with classic KD, however an unbalanced weighing of ingredients (Foster et al., 2003; Kossoff et al., 2003). Moreover, MAD KD does not follow a strict ketogenic ratio and contains protein, fluid, and calorie restrictions (Kang et al., 2007; Kossoff et al., 2007). Despite the similarity of KD efficacy in lowering the glycemic index and other benedficial effects (Pfeifer and Thiele, 2005), the molecular mechanistic action remains to be determined.

The common belief is that increasing dietary fat intake perpetually leads to the fatty liver (Lundsgaard et al., 2019), however, several studies have acknowledged that classic KD improves hepatic lipid profile and alleviates NAFLD (Mardinoglu et al., 2018; Hyde et al., 2019; Watanabe et al., 2020; Zhu et al., 2022). Indeed, Luukkonen *et al.* examined the effects of KD for 6 days on hepatic steatosis and found reduced liver fat content and hepatic insulin resistance in NAFLD patients (Luukkonen et al., 2020). Another study with NAFLD patients found improvement in steatosis, necroinflammation, and fibrosis following KD for 6 months (Tendler et al., 2007). Further, 2 weeks intervention with a KD in obese patients with NAFLD showed a concomitant reduction in DNL and liver fat (Mardinoglu et al., 2018) (Leonetti et al., 2015; Bruci et al., 2020). Subjecting NAFLD patients to a spanish ketogenic mediterranean diet for 12 weeks showed a considerable improvement in steatosis score, AST, and ALT levels (Pérez-Guisado and Muñoz-Serrano, 2011). Another study on 24 patients with obesity placed on a very low-calorie ketogenic diet for 6 months showed a significant improvement in serum liver function markers and triglycerides (Bruci et al., 2020). Despite the beneficial effects, some studies have raised safety concerns for KDs, particularly when subjected to high-fat content. For example, patients subjected to a very-low calorie ketogenic diet showed increased serum cholesterol (Saslow et al., 2017) and liver function markers such as AST and ALT (Colica et al., 2017; Schwenger et al., 2018). Recently intermittent fasting, a strong inducer of ketogenesis, gained attention in effectively treating the NAFLD patients (Anton et al., 2018). Intermittent fasting in the form of time-restricted fasting, periodic fasting or calorie restriction significantly reduce the liver lipid accumulation and improve biochemical liver function indices such as AST and ALT levels (Browning et al., 2011; Wilhelmi de Toledo et al., 2019).

KD might protect from NAFLD through several mechanisms. First, the consumption of a KD induces a metabolic state that resembles fasting which results in weight loss and improvement in metabolic homeostasis (Bahr et al., 2020). Secondly, the anti-steatotic effects of KD are due to the hydrolysis of hepatic lipids and their diversion to ketogenic pathway, which is associated with lower serum insulin levels and hepatic citrate synthase flux, respectively (Luukkonen et al., 2020). KD activates PPARα, a critical downstream target of FGF21, increasing fatty acid β-oxidation (Badman et al., 2009). Third, KD decreases hepatic DNL and even fatty acid synthesis by suppressing stearoyl-CoA desaturase activity (Kennedy et al., 2007). Fourth, KD promotes mitochondrial biogenesis and function by inducing the expression of peroxisome proliferator-activated receptor γ coactivator 1α (PGC1α) in the liver (Ahola-Erkkilä et al., 2010; Jornayvaz et al., 2010). At central levels, KD affects satiety, leading to decreased food intake. KD also inhibit oxidative stress, and inflammation via inhibition of NLRP3, activation of GPRs and histone acetylation (Shimazu et al., 2013; Gibson et al., 2015; Youm et al., 2015). Thus, KD in the form of calorie restriction and macronutrient distribution is effective in the management of NAFLD, although the molecular mechanisms underlying these observed effects are yet to be uncovered.

## Conclusion

NAFLD affects nearly one-third of the population worldwide. The transition from simple steatosis to advanced stages of NAFLD depends on the accumulation of excess lipids due to an imbalance in lipid uptake and disposal. An essential lipid disposal mechanism, hepatic ketogenesis progressively declines as NAFLD severity worsens. Recent studies demonstrate a causal role of impaired hepatic ketogenesis in NAFLD pathogenesis. Accumulating evidence suggests that hepatic ketogenesis alleviates simple steatosis and NAFLD progression. Therefore, defining the mechanisms of hepatic ketogenesis is of great interest in identifying novel therapeutic targets in metabolic diseases, particularly NAFLD.
